# Supplementary material for: WordCommentsAnalyzer: A windows software tool for qualitative research
Source: F1000Res. 2018 Sep 4;7:536. Originally published 2018 May 3. [Version 2] doi: 10.12688/f1000research.14819.2 (PMC6137411; doi:10.12688/f1000research.14819.2)
Supplement: Supplementary file 3 [file f1000research-7-17614-s0002.tgz › b2a79496-4ada-47c4-9682-a99ce867836d.zip!/Eight Iranian Health Journals/Yafteh _62.docx]

© Yafteh  . This work is licensed under CC BY 4.0 (https://creativecommons.org/licenses/by/4.0/).

Volume & Issue: دوره 5 - شماره 3

Title: بررسی ایمنی سلولی در بیماران آسبستوزیس و کارگرانی که در تماس باآسبس هستند

http://yafte.lums.ac.ir/article-1-980-fa.html

مقدمه : تحقیقات متعدد ثابت نموده مزوتلیومای بدخیم در کارگرانیکه در تماس با ذرات آسبس هستند به طور واضح و فراوان دیده می شود. هدف از این مطالعه بررسی میزان پاسخهای ایمنی در کارگرانیکه در تماس با آسبس هستند و مقایسه آن با افراد نرمال بود . مواد و روشها : در این مطالعه 7 بیمار مبتلا به آسبستوزیس، 73 کارگر در تماس با فیبرهای آسبس به عنوان گروه مورد و 59 کارگر بدون تماس با آسبس به عنوان کنترل از دو کارخانه فارسیت درود و تهران مورد بررسی قرار گرفتند. جواب ایمنی سلولی نسبت به PHA و درصد T سل ها و TG سل ها (T ساپرسورسل ها) در 7 بیمار , 16 کارگر در تماس با آسبس و 23 کنترل انجام شد. همچنین جواب آلرژی تأخیری بوسیله آنتی ژنهای P‏PD و کاندیدین در 57 کارگر و 29 کنترل تست شد. یافته ها : درکشت لنفوسیتها نسبت به PHA تغییر آماری مشاهده نشد؛ ولی درصد T سلها در بیماران ( 14±34) و کارگران در تماس با آسبس (16±34 ) در مقایسه با کنترل (21±59 ) تقلیل یافته بود؛ در صورتیکه TG سلها در بیماران (16±15) و کارگران در تماس با آسبس (8±19) نسبت به کنترل (3±9) افزایش یافته بود (001/0p<). کارگران در تماس با آسبس 61% نسبت به PPD و 65 % نسبت به کاندیدین جواب مثبت دادند که در مقایسه با کنترل ( 83% با PPD و 88% نسبت به کاندیدین) کاهش یافته بود(001/0p<). نتیجه گیری : این نتایج نشان میدهد که در بیماران آسبستوزیس و کارگرانی که بیشتر از 11 سال با آسبس تماس دارند، اختلال ایمنی سلولی وجود دارد که معمولاً این اختلال سلولی بعد از تماس زیاد با فیبرهای آسبس و قبل از بروز علائم فیبروزی قابل مشاهده، با رادیوگرافی قابل تشخیص است.

Title: مهار درد حاد و مزمن با عصاره آبی علف چای (Hypericum perforatum L.) در موش صحرایی نر

http://yafte.lums.ac.ir/article-1-981-fa.html

مقدمه : گیاه هاپیریکوم پرفوراتوم در پژوهشکده گیاهان دارویی دانشگاه شهید بهشتی کد گذاری گردید و عصاره گیری با استفاده از روش جوشاندن (decoction) انجام شد. گزارشات فراوانی راجع به اثرات ضدافسردگی هایپریکوم پرفوراتوم وجود دارد؛ اما در رابطه با اثرات ضد دردی خانواده هایپریکوم (به ویژه هایپریکوم پرفوراتوم) مطالعات کمی انجام شده است. هدف از این تحقیق بررسی اثرات مهاری عصاره آبی علف چای بر درد حاد (حرارتی و شیمیایی) و درد مزمن (شیمیایی) می باشد. مواد و روشها : در این مطالعه تجربی از 70 عدد موش صحرایی نرSprague-Dawely در محدوده وزنی 200-230 گرم برای تمام آزمایش ها و برای تعیین دوز کشنده از 50 عدد موش سوری با محدوده وزنی 37-34 گرم انجام شد. برای اندازه گیری درد از دو روش آزمایش فرمالین (مدل درد حاد و مزمن شیمیایی) و آزمایش Tail-flick ( درد حاد حرارتی) استفاده گردید. اثرات ضد دردی تجویز داخل صفاقی عصاره آبی در سه دوز(200، 400 وmg/kg 800) بررسی شد. اثرات ضد دردی عصاره هایپریکوم با سدیم سالیسیلات به عنوان شاهد مثبت مقایسه گردید. محدوده دوز کشنده LD50 حدود mg/kg 6000 تعیین گردید. آنالیز آماری بر حسب مورد، paired t-test، unpaired و ANOVA انجام شد. یافته ها : نتایج آزمایشات نشان می دهد که تجویز داخل صفاقی سدیم سالیسیلات mg/kg)300 ) بر زمان تاخیر آزمون Tail flick اثری نداشت؛ در حالیکه عصاره آبی در تمامی دوزهای مصرفی باعث افزایش زمان تاخیر آزمون گردید. همچنین عصاره آبی در تمامی دوزهای مصرفی بی دردی حیوان را در هر دو فاز آزمون فرمالین افزایش داد؛ اما سدیم سالیسیلات فقط بر فاز دوم آزمون فرمالین مؤثر بود. نتیجه گیری: نتایج نشان می دهند که عصاره آبی هایپریکوم بر مدل های درد حاد (حرارتی وشیمیایی) و درد مزمن (شیمیایی) مؤثرمی باشد و همچنین می توان اثرات ضددردی محیطی و مرکزی برای عصاره قائل شد.

Title: بررسی اثر آنتی باکتریال عصاره های الف ، جوشن ، همیشه سبز و سماق لری بر روی تعدادی از باکتریهای گرم مثبت وگرم منفی

http://yafte.lums.ac.ir/article-1-982-fa.html

مقدمه : با توجه به افزایش روز افزون مقاومت باکتریها نسبت به آنتی بیوتیکها و عوارض جانبی آنها استفاده از گیاهان داروئی و عصاره های گیاهی مورد توجه زیادی قرار گرفته اند . در این پژوهش اثرات آنتی باکتریال عصاره های سماق لرستان ، الف و درمنه (جوشن ) و همیشه سبز بر روی شش باکتری گرم منفی و گرم مثبت استاندارد مورد ارزیابی قرار گرفتند ، مواد و روشها : برای انجام این پژوهش آزمایشی ابتدا برگ یا میوه تازه گیاه در سایه خشک و تبدیل به پودر شد، سپس در چند مرحله عصاره آن درحلالهای آلی بدست آمد. با تبخیر نمودن حلال ، عصاره در سرم فیزیولوژی استریل بازسازی شده آزمایش دیسک دیفیوژن (DD) و تعیین حداقل غلظت مهار کنندگی (MIC) به روش برات میکرودایلوشن انجام گرفت. پس از آماده شدن حداقل غلظت کشندگی باکتریها (MBC )، مورد استفاده قرار گرفتند . یافته ها : نتایج نشان داد که عصاره سماق لری (وحشی لرستان ) اثر قوی آنتی باکتریال دارد؛ بطوریکه در رقت بسیار پایین ( μg/ml5MIC=) رشد باسیلوس سروس رامتوقف نمود و در غلظتml /μg78= MBC موجب مرگ این باکتری شد . اثر عصاره سماق بر باکتریهای گرم مثبت بیشتر از گرم منفی بود؛ به عنوان مثال اثر باکتریواستاتیک و باکتریوسیدال این عصاره بر روی انتروکک فکالیس برابر با μg/ml30MBC=MIC= بود .در حالیکه همین اثر برای سودوموناس و اشرشیاکلی μg/ml600 یا بیشتر بود. همچنین عصاره گیاه الف در غلظت های پائین برباسیلوس سروس اثر آنتی باکتریال داشت و در غلظت μg/ml600 بر استاف اپیدرمیدیس و اشرشیاکلی موثر بود .اثر آنتی باکتریال عصاره همیشه سبز بر باکتریهای مورد آزمایش بسیار کم و یا در غلظت مورد آزمایش بی اثر بود . عصاره گیاه جوشن نیز به همین ترتیب بر بعضی از باکتریهای مورد آزمایش موثر بود . نتیجه گیری : با توجه به نتایج فوق می توان امیدوار بود که عصاره سماق را می توان در درمان بعضی از باکتریهای مقاوم مانند انتروکک فکالیس استفاده نمود .همچنین در صنایع غذائی نیز بعنوان محافظت کننده مواد غذائی قابل استفاده می باشند .

Title: اثر اعتیاد به مورفین والدین بر غلظت گلوتامات پایه و تحریکی ناحیه شکنج دندانه‌ای هیپوکامپ فرزندان موش صحرایی

http://yafte.lums.ac.ir/article-1-983-fa.html

مقدمه: شواهد نشان می‌دهد که اعتیاد یکی از والدین به مورفین منجر به اختلال در فرآیند یادگیری و حافظه فرزندان می شود. با توجه به نقش گلوتامات در ناحیه شکنج دندانه‌ای هیپوکامپ بر یادگیری، در این مطالعه اثر اعتیاد یکی از والدین بر غلظت گلوتامات پایه و تحریکی ناحیه شکنج دندانه‌ای فرزندان موش صحرایی مورد بررسی قرار گرفت. مواد و روشها: در این مطالعه تجربی 20 سرموش صحرایی ماده و8 سرموش صحرایی نر از نژاد ویستار با مصرف خوراکی مورفین با دوز 32 میلی گرم / کیلوگرم، 2بار در روز معتاد شدند. به منظور انجام جفت گیری 20 سر موش ماده معتاد با 8 سر موش نر غیر معتاد و 28 موش نر معتاد با 20 سر موش ماده غیر معتاد در قفس‌های جداگانه‌ای قرار گرفتند. پس از رسیدن فرزندان به سن بلوغ مایع خارج سلولی ناحیه شکنج دندانه‌ای آنها در حالت پایه و تحریک الکتریکی ( بافرکانس 10 هرتز و جریان 700 میکرو آمپر) مسیر نفوذی، به روش میکرو دیالیز استخراج و با استفاده از دستگاه HPLC وردیاب فلورسنت اندازه‌گیری شد. نتایج به صورت میانگین± خطای معیار بیان گردید و نتایج به روش آنالیز واریانس دو طرفه مقایسه شدند. یافته ها : در فرزندان نر گروه مادر معتاد، غلظت گلوتامات پایه و تحریکی ناحیه شکنج دندانه‌ای هیپوکامپ به ترتیب 04/0±67/0؛ 1/0±11/1 ودر فرزندان ماده به ترتیب06/0±47/0؛ 05/0±88/0نانومول بود( 5 =n). مقادیر غلظت های مشابه درگروه پدر معتاد، به ترتیب 15/0±06/1؛ 2/0± 03/2و 05/0±9/0 ؛ 11/0±75/1 نانومول بود( 5 =n). نتایج نشان داد که در فرزندان هر دو گروه مادر معتاد و پدر معتاد، غلظت گلوتامات خارج سلولی پایه و تحریکی ناحیه شکنج دندانه‌ای نسبت به گروه شاهد حود به طور معنی داری کاهش یافته است. در گروه مادر معتاد میزان گلوتامات پایه و تحریکی در ناحیه شکنج دندانه‌ای در فرزندان ماده کمتر از فرزندان نر بود. میزان گلوتامات پایه و احریکی ناحیه شکنج دندانه ای فرزندان در گروه مادر معتاد به طور معنی داری کمتر از گروه پدر معتاد بود. نتیجه گیری : با توجه به نتایج این مطالعه می‌توان نتیجه‌گیری کرد که مصرف مورفین می‌تواند منجربه کاهش غلظت گلوتامات مایع خارج سلولی ناحیه شکنج دندانه‌ای در فرزندان گردد که به نوبه خود می‌تواند موجب اختلالاتی در روند‌ حافظه و یادگیری شود. بنابر این آگاه کردن افکار عمومی از عواقب اثر اعتیاد بر فرزندان بایستی بیشتر مورد توجه قرارگیرد .

Title: اثر سویا بر روی لیپیدهای سرم در خانم های یائسه مبتلا به هیپرکلسترونمی مراجعه کننده به بیمارستان عسلیان خرم آباد

http://yafte.lums.ac.ir/article-1-984-fa.html

مقدمه: یکی از مشکلات عمده خانم ها درسنین بعد از یایسگی بیماری قلبی ـ عروقی یاCVD است که باعث افزایش مرگ و میر خانم ها در زمان یایسگی می شود. یک علت عمده بیماریهای قلبی ـ عروقی آترواسکروز است و چنین فرض شده است که استروژن باعث محافظت زنان در برابر آترواسکروز می شود. سویا بدلیل دارا بودن ایزوفلان هایی چون ژنیستین و دایدزین که جزء استروژنهای گیاهی طبقه بندی شده است. به عنوان یک منبع عمده استروژن گیاهی شناخته شده است. هدف از انجام این پژوهش بررسی اثر سویا بر روی لیپیدهای سرم در خانم های یائسه مبتلا به هیپرکلسترونمی بود. مواد و روشها: این مطالعه، یک کار آزمایی بالینی است که بر روی 100 خانم یائسه دچار افزایش کلسترول پلاسما مراجعه کننده به بیمارستان زنان عسلیان خرم آباد در سال 1381 انجام گرفت. این افراد به طور تصادفی به دو گروه 50 نفری تقسیم شدند که به گروه آزمایش سویا به صورت کپسولهای mg 500 با دوز gr/day2 دوبار در روز و به گروه گواه کپسولهای حاوی ماده بی اثر داده شد. 12 هفته پس از مصرف سویا از هر دو گروه نمونه خون گرفته و به آزمایشگاه ارسال شد. اطلاعات بدست آمده با استفاده از نرم افزار SPSS تجزیه و تحلیل شدند. یافته ها: نتایج حاصله نشان داد که بین میانگین کلسترول سرم قبل از درمان و 12 هفته بعد از درمان اختلاف معنی داری وجود ندارد (p=NS). بین سطح کلسترول گروه آزمایش قبل و بعد از مصرف سویا تغییر معنی داری حاصل نشد (p=NS). همچنین بین سطح کلسترول آزمایش و گروه گواه تفاوت معنی داری مشاهده نشد (p=NS). نتیجه گیری : با توجه به نتایج فوق سویا نمی تواند جایگزین مناسبی برای استروژن در درمان یائسگی باشد.

Title: بررسی موارد ابتلا به سرخک در کودکان شهرستان خرم آباد

http://yafte.lums.ac.ir/article-1-985-fa.html

مقدمه : سرخک بیماری است که به راحتی از شخصی به شخصی دیگر سرایت می کند.گرچه امروزه تعداد موارد ابتلا به سرخک به نحو قابل ملاحظه ای کاهش یافته؛ اما هنوز هم بیماری شایعی می باشد. هدف از انجام این مطالعه بررسی موارد ابتلا به سرخک در کودکان شهرستان خرم آباد بود. مواد و روشها : این مطالعه بصورت توصیفی ـ مقطعی انجام شد .کلیه کودکانی که در بیمارستان شهید مدنی خرم آباد با تشخیص سرخک بستری شده بودند (124 بیمار) مورد بررسی قرار گرفتند. متغییرهای مورد مطالعه شامل سن, جنس, ماه و فصل بستری بود. اطلاعات توسط پرسشنامه جمع آوری و بوسیله نرم افزار Spss مورد تجزیه و تحلیل آماری قرار گرفتند. یافته ها : از 124 بیمار تحت بررسی , 78 نفر(9 /62%) پسر و 46 نفر (1/27%) دختر بودند. بیشتر موارد در سنین زیر 9 ماهگی (6/47%) و 7-6 سالگی (9/37%) روی داد. بین سن ابتلا به سرخک و همچنین بین جنس و ابتلا به سرخک رابطه آماری معنی داری وجود داشت. (05/0p<). بیماران بستری شده طی فروردین تا اسفند 1381, 31 نفر ( 25%)و طی فروردین تا تیر 1382, 93 نفر( 75%) بودند. نتیجه گیری : در این مطالعه بیشترین میزان بروز سرخک قبل از 9 ماهگی و پس از 6 سالگی بودو این در حالی است که طبق دستورالعمل وزارت بهداشت ایران، واکسن سرخک طی دو دوز شامل9 ماهگی و 15 ماهگی تزریق می گردد. بنابراین توصیه می شود در صورت تائید نتایج این مطالعه در سایر شهرستانها، تغییرات اساسی در برنامه واکسیناسیون ایران انجام شود.

Title: مقایسه اثرات hCG و سولفات منیزیم در پیشگیری

http://yafte.lums.ac.ir/article-1-986-fa.html

مقدمه : با توجه به شیوع بالای زایمان زودرس و عوارض شناخته شده آن و اینکه گیرنده های hCG در میومتر رحم انسان وجود دارد و hCG سبب مهار انقباضات میومتر در خارج از بدن می شود، در صدد برآمدیم تا اثرات درمان hCG را در زایمان زودرس بررسی کرده و همچنین با سولفات منیزیم که درمان متداول آن است نیز مقایسه کنیم مواد و روشها: این کار آزمایی بالینی (Clinical trial ) بر روی زنان حامله بین 35-25 هفته ای مبتلا به انقباضات رحمی با کیسه آب سالم و دیلاتاسیون سرویکس کمتر از 4 سانتی متر بستری در زایشگاههای شهرستان خرم آباد بین سالهای 79 لغایت 81 صورت پذیرفت. در این بررسی 101 زن حامله بستری در بیمارستانهای شهر خرم آباد به 2 گروه تقسیم شدند. گروه مورد (50 نفر ) که تحت درمان با hCG به صورت 5000 واحد در شروع و سپس 10000 واحد در 500 سی سی سرم قندی ریخته و با سرعت 20 قطره در دقیقه به بیماران داده شد و گروه شاهد (51 نفر) تحت درمان متداول با سولفات منیزیم قرار گرفتند. درمان تا قطع انقباضات رحمی ادامه پیدا می کرد و عوارض جنینی و مادری حین درمان نیز ثبت می شد. بیماران 24 ساعت بعد از قطع درمان در بیمارستان و سپس بعد از ترخیص در مراجعاتی که به درمانگاه و مطب داشتند تا زمان زایمان پی گیری شدند. یافته ها : گروهها به لحاظ سن مادر , سن حاملگی و گراویدیتی تقریبا“ مشابه بودند میزان تاخیر در گروه hCG 68/22 روز و در گروه سولفات منیزیم 57/24 روز بود. در گروه hCG 9 نوزاد (18 درصد) و در گروه سولفات منیزیم نیز 9 نوزاد (8/17 درصد) در بخش مراقبت های ویژه نوزادان (NICU) بستری شدند. میانگین وزن در گروه hCG 2334 گرم و در گروه سولفات منیزیم 2287 گرم بود که اختلاف معنی دار نداشتند (p=NS). در هیچکدام از گروهها دارو قطع نشد؛ ولی میزان شکایت و عوارض در گروه سولفات منیزیم صد درصد بوده و در گروه hCG صفر بود (001/0p<) نتیجه گیری: با توجه به مشابه بودن تاثیر هر دو دارو در تاخیر زایمانی و سرکوب انقباضات رحمی و میانگین وزن هنگام تولد و بدون عارضه بدون hCG، استفاده از این دارو در پیشگیری از زایمان زودرس به عنوان جانشین سولفات منیزیم منطقی به نظر می رسد و hCG یک کاندیدا مناسب درمان زایمان زودرس می باشد.

Title: بررسی رشد و توسعه لارو اکینوکوکوس گرانولوزوس

http://yafte.lums.ac.ir/article-1-987-fa.html

مقدمه: کیست هیداتید از بیماری های انگلی مشترک انسان، دام، گوشتخواران اهلی و وحشی می باشد. این بیماری در انسان موجب ضایعات و آسیبهای عضو شده و گاهی منجر به مرگ می شود . هدف از انجام این مطالعه بررسی رشد کیست هیداتید ثانویه در حیوانات آزمایشگاهی، تعیین حساسیت آنها، تهیه مقاطع بافتی و چگونگی رشد و تشکیل مراحل کیست هیداتید ثانویه بود. مواد و روشها : به منظور بررسی رشد کیست هیداتید ثانویه و تعیین حساسیت موش سوری و هامستر سفید، تعداد 36 سر موش سوری و 27 سر هامستر سفید ، با ترزیق داخل صفاقی لارو اکینوکوکوس گرانولوزوس گوسفندی مورد ارزیابی قرار گرفتند؛ سپس به مدت 9 ماه ، ماهیانه تعداد معینی از حیوانات کالبدگشایی شده و محوطه صفاق ، کبد ، ریه ، کلیه و طحال از نظر آلودگی به کیست ، مورد بررسی قرار گرفتند. یافته ها : در این بررسی کیست هیداتید ثانویه در موش سوری از ماه سوم تشکیل گردید و در ماههای بعد بخصوص ماه هشتم و نهم در کلیه موشها کیست هیداتید به تعداد زیاد رشد کرد؛ بطوری که در دو سر از موشهایی که در ماه نهم کالبدگشایی شدند، تعداد 8 و 14 کیست در محوطه صفاقی آنان مشاهده گردید. نتیجه گیری : کلیه کیست ها غیر بارور و استریل بوده و با افرایش مدت آلودگی بر تعداد موش های مثبت و تعداد کیست ها افزوده گردید. بررسی مقاطع بافتی ، دیواره کیست و واکنش سلولهای دفاعی را نشان داد. موش سوری حیوان مناسبی برای رشد کیست هیداتید ثانویه می باشد و از ماه سوم آلودگی کیستها قابل مشاهده است.

Title: مقایسه عوارض ناشی از آیودی های cuT 380 Aو cu safe 300 در سه ماهه اول بعد از جایگذاری

http://yafte.lums.ac.ir/article-1-988-fa.html

مقدمه :.مطالعات نشان داده است میزان بروز عوارض در انواع مختلف آیودی متفاوت است و هر چه اندازه آیو دی کوچکتر باشد، عوارضی مثل خونریزی و درد کمتر است. هدف از انجام این مطالعه تعیین و مقایسه عوارضی مثل خونریزی،درد و دفع خود به خود آیودی در استفاده کنندگان آیودی های cuT 380A و cu Safe 300 در سه ماهه اول بعد از جایگذاری بود. مواد و روشها : پژوهش حاضر یک کارآزمایی بالینی است که بر روی کلیه خانم های متقاضی آیو دی مراجعه کننده (243) به مراکز بهداشتی درمانی شهر خرم آباد طی 9 ماه انجام شد. نمونه گیری به صورت تصادفی ساده انجام شد.اطلاعات از طریق پرسشنامه و با استفاده از مصاحبه و مشاهده جمع آوری گردید. یافته ها : نتایج حاصل از پژوهش نشان داد طی سه ماه شدت و شیوع درد و دفع خودبخود در گروه cuT 380 A بیشتر از گروه cu safe 300 بود و با استفاده از آزمون های آماری تفاوت معنی داری بین دو گروه مشاهده شد؛ ولی از نظر شدت خونریزی بین دو گروه تفاوت معنی داری مشاهده نشد. نتیجه گیری : :مطالعه حاضر نشان می دهد که آیو دی های جدید(CU Safe 300) که در اندازه ای کوچکتر و سازگار با انقباضات رحم ساخته شده اند، کم عارضه تر بوده و بسیار مورد پذیرش استفاده کنندگان واقع شده اند بنابراین توصیه می شود در صورت امکان استفاده از این آیودی جدید نیز در مراکز بهداشتی درمانی گسترش یابد.

Title: بررسی میزان موفقیت TUI در درمان پروستاتیت مزمن مقاوم

http://yafte.lums.ac.ir/article-1-989-fa.html

مقدمه: پروستاتیت مزمن بیماری مزاحم, آزار دهنده و شایعی است که بسیاری به آن مبتلا هستند. درمان این بیماری مشکل است و اغلب به خاطر عودهای مکرر آن مشکلات پیچیده ای برای بیمار و پزشک معالج بدنبال دارد. مواد و روشها: در این مطالعه مداخله ای از بیمارانی که مبتلا به پروستاتیت مزمن بودند و به درمان های داروئی رایج پاسخ رضایت بخشی نمی دادند، بیست نفر به طور تصادفی انتخاب و کاندید عمل TUI شدند. قبل از عمل اطلاعاتی در خصوص مشخصات فرد وعلائم بیماری آنها در فرم مخصوص ثبت می شد پس از توضیح کامل نحوه عمل جراحی و عوارض احتمالی عمل جراحی و کسب رضایت, بیمار تحت عمل جراحی TUI قرار می گرفت. حداقل شش هفته بعد از عمل و در پیگیری های بعدی از بیمار علائم بیماری قبل از عمل (مطابق فرم قبل از عمل) ثبت و مقایسه می شد. معیار بررسی تغییرات علائم بارز پروستاتیت مزمن شامل: شب شاشی, تکرر ادرار, سوزش ادرار, کالیبر و جریان غیر طبیعی ادرار, در انتهای ادرار قطره قطره آمدن ادرار, درد در حین مقاربت, برگشت انزال بداخل مثانه (ازعوارض احتمالی عمل), اختلال در تمایل جنسی بود. اطلاعات بدست آمده با استفاده از آزمون Wilcoxon مورد تجزیه و تحلیل قرار گرفتند. یافته ها: این بررسی نشان داد بیماری که تحت درمان با TUI قرار گرفتند به میزان قابل توجهی نسبت به قبل از عمل بهبودی پیدا کردند. علائم بارز پروستاتیت شامل شب شاشی تکرر ادرار و.. به طور معنی داری (P<0/05) نسبت به قبل از عمل کاهش پیدا کرده بود. میزان رضایت بیماران کاملا“ مشهود بود و نیاز بدرمان داروئی به میزان قابل توجهی کاهش پیدا کرد. نتیجه گیری : توصیه می شود انجام TUI به عنوان یک آلترناتیو درمانی مناسب برای مبتلایان به پروستاتیت مزمن مقاوم بدرمان مدریکال مورد توجه قرار گیرد .
